# Epidemiological characteristics of patients with idiopathic normal pressure hydrocephalus in Japan: analysis of the treatment

**DOI:** 10.1186/2045-8118-12-S1-O39

**Published:** 2015-09-18

**Authors:** Madoka Nakajima, Masakazu Miyajima, Chihiro Akiba, Ikuko Ogino, Hajime Arai

**Affiliations:** 1 Juntendo University School of Medicine, Japan

## Introduction

To clarify the epidemiological and clinical characteristics of idiopathic normal pressure hydrocephalus (iNPH) in Japan, a nationwide epidemiological survey was conducted, focusing on treatment analysis.

## Methods

Study participants were selected according to hospital bed capacity, using a random sampling method. In a primary survey, the number of iNPH patients in each hospital department during the year 2012 was estimated for stratification of institutions and next, in a secondary survey, selected patients provided detailed information, determining the clinical and epidemiological features of interest.

## Results

In the primary survey 1804 clinical departments responded (recovery rate: 42.7% ) for 3079 individuals, including 1815 individuals who underwent shunt surgery. The estimated annual number of patients receiving treatment was 13,000 (95% CI: 10000 to 16000). The estimated prevalence rate for hospital-based patients in 2012 was 10.2 individuals per 100,000.

Analyses were conducted on 1495 respondents (885 males (59.2%): age 77.9±6.30(SD) years; and 610 females (40.8%): age 78.0±6.41 years. Results showed 992 iNPH patients (66.1%) treated with shunt surgery, 547 patients (77.6±6.12yo) with lumboperitoneal (LPS), 428 patients (76.51±6.19yo) with ventriculoperitoneal (VPS) and 17 with vetriculoatrial (VAS) shunting. Four patients having both LPS and VPS.

In 120 out of 992 shunted patients were recorded complications (12.1%), including 77 out of 547 patients with LPS and 43 out 428 patients with VPS, without statistically significant difference between the two subgroups (p=0.092).

Therapeutic efficacy exceeded 90% with improvement of 1 point or higher on the modified Rankin Scale (mRS) in 56%. Age and Alzheimer's Disease were factors with a negative impact on the therapeutic prognosis, with odds ratio of 1.029 95%CI (1.013-1.047) p=0.001, and 1.365 95% CI (1.008-1.849) p=0.044, respectively. They had significant impact on the prognosis of improvement according to mRS.

## Conclusions

We reported hospital based survey study in Japan, providing the main characteristics of iNPH epidemiology and management for 2012.
